# Supplementary material for: Training Optimization for Gate-Model Quantum Neural Networks
Source: Sci Rep. 2019 Sep 3;9:12679. doi: 10.1038/s41598-019-48892-w (PMC6722103; doi:10.1038/s41598-019-48892-w)
Supplement: Supplementary file 1 — Supplemental Information [file 41598_2019_48892_MOESM1_ESM.pdf]

Training Optimization for Gate-Model Quantum Neural Networks

Laszlo Gyongyosi[1,2,3,*] **and Sandor Imre**[2]

[1]School of Electronics and Computer Science, University of Southampton, Southampton, SO17 1BJ, UK
[2]Department of Networked Systems and Services, Budapest University of Technology and Economics, Budapest, H-1117 Hungary
[3]MTA-BME Information Systems Research Group, Hungarian Academy of Sciences, Budapest, H-1051 Hungary
[*]l.gyongyosi@soton.ac.uk

ABSTRACT

Gate-based quantum computations represent an essential to realize near-term quantum computer architectures. A gate-model quantum neural network (QNN) is a QNN implemented on a gate-model quantum computer, realized via a set of unitaries with associated gate parameters. Here, we define a training optimization procedure for gate-model QNNs. By deriving the environmental attributes of the gate-model quantum network, we prove the constraint-based learning models. We show that the optimal learning procedures are different if side information is available in different directions, and if side information is accessible about the previous running sequences of the gate-model QNN. The results are particularly convenient for gate-model quantum computer implementations.

A Appendix

A.1 Abbreviations

AI Artificial Intelligence

DAG Directed Acyclic Graph

QG Quantum Gate structure of a gate-model quantum computer

QNN Quantum Neural Network

RQNN Recurrent Quantum Neural Network

A.2 Notations

The notations of the manuscript are summarized in Table A.1.

Table A.1. Summary of notations.

Notation	*Description*
QNN_{QG}	Quantum neural network implemented on a gate-model quantum computer with a quantum gate structure QG.
RQNN_{QG}	Recurrent quantum neural network implemented on a gate-model quantum computer with a quantum gate structure QG.
$U_i(\theta_i)$	An i-th unitary gate, $U_i(\theta_i) = \exp(-i\theta_i P)$, where P is a generalized Pauli operator formulated by a tensor product of Pauli operators $\{X, Y, Z\}$, while θ_i is referred to as the gate parameter associated to $U_i(\theta_i)$.
$U_j(\theta_{ij})$	Selection of θ_j for the unitary U_j to realize the operation $U_i(\theta_i)U_j(\theta_j)$, i.e., the application of $U_j(\theta_j)$ on the output of $U_i(\theta_i)$ at a particular gate parameter θ_j.
$U(\vec{\theta})$	Unitary operator, $U(\vec{\theta}) = U_L(\theta_L)U_{L-1}(\theta_{L-1})\ldots U_1(\theta_1)$, where $U_i(\theta_i)$ identifies an i-th unitary gate.

$\vec{\theta}$	A collection of gate parameters of the L unitaries, $\vec{\theta} = \theta_L, \theta_{L-1}, \ldots, \theta_1$.
$\|\psi, \varphi\rangle$	Input system, where $\|\psi\rangle = \|z\rangle$ is a computational basis state, where z is an n-length string, while the $(n+1)$-th quantum state initialized as $\|\varphi\rangle = \|1\rangle$, and is referred to as the readout quantum state.
$\|Y\rangle$	An $(n+1)$-length output quantum system of the gate-model quantum neural network.
z	An n-length string, $z = z_1 z_2 \ldots z_n$, where z_i represents a classical bit, $z_i \in \{-1, 1\}$.
$f(\theta)$	Objective function.
$l(z)$	Binary label of string z, $l(z) \in \{-1, 1\}$.
$\tilde{l}$	Predicted value of the binary label $l(z) \in \{-1, 1\}$ of string z, $\tilde{l}(z) = \langle z, 1\|(U(\vec{\theta}))^{\dagger} Y_{n+1} U(\vec{\theta})\|z, 1\rangle$.
$\Delta(\tilde{l}(z))$	Difference of the $\tilde{l}(z)$ predicted value of the binary label $l(z) \in \{-1, 1\}$ of the input string z, defined as $\Delta(\tilde{l}(z)) = \|l(z) - \tilde{l}(z)\|$, where $\tilde{l} \in [-1, 1]$.
Y_{n+1}	Measured Pauli operator on the $\|\varphi\rangle$ readout quantum state, $Y_{n+1} \in \{-1, 1\}$.
$\|Y\rangle^{(r)}$	An output system realization, $r = 1, \ldots, R$, where R is the total number of output instances.
$\mathscr{S}_T$	Training set, formulated via N input strings and labels, $\mathscr{S}_T = \{z_i, l(z_i), i = 1, \ldots, N\}$.
$\mathscr{C}$	Constraint machine.
$\mathscr{D}$	Diffusion machine.
$\mathscr{F}$	Functional space.
$\mathscr{G}$	Environmental graph, $\mathscr{G} = (V, S)$. A directed acyclic graph (DAG), with a set V of vertexes, and a set S of arcs.
V	Set of vertexes in the $\mathscr{G}$ environmental graph.
S	Set of arcs in the $\mathscr{G}$ environmental graph.
v	A vertex of V the $\mathscr{G}$ environmental graph.
$\Gamma(v)$	Children set of v the $\mathscr{G}$ environmental graph.
$\|\Gamma(v)\|$	Cardinality of set $\Gamma(v)$.
$\langle x \rangle$	An identifier.
$\mathscr{X}$	Perceptual space.
$\mathscr{Z}$	Mapped space.
x	An element (vector) of the perceptual space $\mathscr{X} \subset \mathbb{C}^d$.
$\diamondsuit$	Symbol of missing features.
$\mathscr{X}_0$	Initial perceptual space, $\mathscr{X}_0 = \mathscr{X} \bigcup \{\diamondsuit\}$.
$\mathscr{I}$	Individual space, $\mathscr{I} = V \times \mathscr{X}_0$.
$f_{\mathscr{P}}$	A perceptual map, $f_{\mathscr{P}} : \tilde{V} \to \mathscr{X} : x = f_{\mathscr{P}}(v)$, where $\tilde{V}$ is a subset V in the $\mathscr{G}$ environmental graph.

ι	An individual of the individual space $\mathscr{I}$, $\iota = \Upsilon x + \neg\Upsilon v$, where $+$ is the sum operator in $\mathbb{C}^d$, while Υ is a constraint as $\Upsilon : (v \in \tilde{V}) \vee (x \in \mathscr{X} \setminus \mathscr{X}_0)$.
C_{ι^*}, C_ι	Constraints.
$\chi(\cdot)$	Compact constraint.
$\mathscr{S}_{in}$	Input space.
$\mathscr{U}$	Space of unitaries.
$\mathscr{Y}$	Output space.
$\mathscr{G}_{\text{QNN}_{QG}}$	Environmental graph of a QNN_{QG}.
$\mathscr{G}_{\text{RQNN}_{QG}}$	Environmental graph of an RQNN_{QG}.
v_{U_i}	A vertex associated to the unitary $U_i(\theta_i)$ in the environmental graph.
v_0	A vertex associated to the input in the environmental graph.
θ_{ij}	Gate parameter, associated to the directed arch s_{ij} between v_{U_i} and v_{U_j}.
$x_{U_i(\theta_i)}$	An element of $\mathscr{X}$ associated to unitary $U_i(\theta_i)$.
x_0	An element of $\mathscr{X}$ associated to the input, $x_0 = \lvert z, 1\rangle$.
$a_{U_i(\theta_i)}$	Parameter defined for a $U_i(\theta_i)$ as $a_{U_i(\theta_i)} = \sum_{h\in\Xi(i)} U_h(\theta_h)\, x_{U_h(\theta_h)} + b_{U_i(\theta_i)}$, where $\Xi(i)$ refers to the parent set of v_{U_i}, $b_{U_i(\theta_i)}$ is the bias relative to v_{U_i}.
$f_\angle(\cdot)$	Topological ordering function on the environmental graph.
$\mathbf{H}$	Hessian matrix.
$f_T(\cdot)$	Transition function.
$F_O(\cdot)$	Output function.
γ	State variable in the mapped space $\mathscr{Z}$, $\gamma \in \mathscr{Z}$.
γ_v	State variable associated to v, $\gamma_v \in \mathscr{Z}$.
$\phi(A)$	Associated function-pair, $\phi(A) = (f_T, F_O)$.
$\lvert\gamma_v\rangle$	System state associated to state variable γ_v.
ζ_v	Constraint on $f_T\left(\text{QNN}_{QG}\right)$ for a QNN_{QG}.
$\wp_v$	Constraint on $F_O\left(\text{QNN}_{QG}\right)$ for a QNN_{QG}.
$\circ$	Composition operator, $(f \circ g)(x) = f(g(x))$.
α	Parameter.
π_v	Compact constraint on $f_T\left(\text{QNN}_{QG}\right)$ and $F_O\left(\text{QNN}_{QG}\right)$.
Λ_v	Constraint on $f_T\left(\text{RQNN}_{QG}\right)$ of RQNN_{QG}.
Ω_v	Constraint on $F_O\left(\text{RQNN}_{QG}\right)$ of RQNN_{QG}.
$\bar{\lambda}(f(x))$	Diffuse constraint for RQNN_{QG}.
H_t	Unit vector for a unitary $U_t(\theta_t)$, $t = 1, \ldots, L-1$, $H_t = x_t + i y_t$, where x_t and y_t are real values.
Z_{t+1}	System state, $Z_{t+1} = U(\vec{\theta})H_t + E x_{t+1}$, where E is a basis vector matrix.
$f_\sigma^{\text{RQNN}_{QG}}(\cdot)$	Function for RQNN_{QG}.

$\|\|_1$	$L1$-norm.
W	An output matrix.
D	Jacobian matrix.
A	Constraint matrix.
$b(x)$	Smooth vector-valued function with compact support.
f^*	Compact function subject to be determined.
$\mathscr{S}_{L(\mathrm{QNN})}$	Non-empty supervised learning set.
ℓ	Differential operator, $\ell = P^{\dagger}P$, where $P^{\dagger}$ is the adjoint of P.
∇^2	Laplacian operator.
$\mathscr{G}(\cdot)$	Green function.
$\mathscr{L}$	Lagrangian.
$\lambda(x)$	Lagrange multiplier.
$H(x)$, Φ, χ_{κ}	Parameters used in the calculation of compact function $f^*(x)$.
$\mathscr{L}(x_0, \tilde{l}(z))$	Loss function.
$\mathscr{A}_{\mathscr{C}}(\mathrm{QNN}_{QG})$	Learning method for $\mathscr{C}\left(\mathrm{QNN}_{QG}\right)$.
$\mathscr{A}_{\mathscr{D}}(\mathrm{RQNN}_{QG})$	Learning method for $\mathscr{D}\left(\mathrm{RQNN}_{QG}\right)$.
$\vec{T}_{\mathscr{G}_{\mathrm{QNN}_{QG}}}$	Topologically sorted node set, $\vec{T}_{\mathscr{G}_{\mathrm{QNN}_{QG}}} = (q_1, \ldots, q_L)$.
$\Xi(k)$	Parents of $k \in V$ in the environmental graph.
$\mathrm{P}^{(r)}(\mathscr{G}_{\mathrm{QNN}_{QG}})$	Post-processing associated to the r-th measurement on $\mathscr{G}_{\mathrm{QNN}_{QG}}$.
$\delta_{U_i(\theta_i)}$	Error associated to unitary $U_i(\theta_i)$ in the environmental graph.
$v_{U_L(\theta_L)}$	Vertex associated to $U_L(\theta_L)$ in the environmental graph.
$\vec{\Delta}\theta_i$	Parameter modification.
$g_{U_i(\theta_i),U_j(\theta_j)}$	Gradient between unitaries.
S_i	Structure from the environmental graph.
$\vec{\delta}_{S_i}$	Error vector associated to structure S_i.
η	Learning parameter.
S'	Children structure.
I	Identity operation.
$\|Q\rangle$	An output realization of RQNN_{QG}.
$\mathbf{H}_{\mathrm{RQNN}_{QG}}$	Hessian matrix of the RQNN_{QG} structure.
$\hbar_{ij,lm}^{\mathrm{RQNN}_{QG}}$	A generic coordinate of the Hessian matrix $\mathbf{H}_{\mathrm{RQNN}_{QG}}$.
$(\delta^{Q}_{U_l(\theta_l),U_i(\theta_i)})^2$	Square error between unitaries $U_l(\theta_l)$ and $U_i(\theta_i)$ at a particular output $\|Q\rangle$ of RQNN_{QG}.
